# Regulation of Cardiac Cav1.2 Channels by Calmodulin

**DOI:** 10.3390/ijms24076409

**Published:** 2023-03-29

**Authors:** Masaki Kameyama, Etsuko Minobe, Dongxue Shao, Jianjun Xu, Qinghua Gao, Liying Hao

**Affiliations:** 1 Department of Physiology, Graduate School of Medical & Dental Sciences, Kagoshima University, Sakura-ga-oka, Kagoshima 890-8544, Japan; 2 Department of Pharmaceutical Toxicology, School of Pharmacy, China Medical University, Shenyang 110012, Chinalyhao@cmu.edu.cn (L.H.)

**Keywords:** Cav1.2, Ca^2+^ channel, calmodulin

## Abstract

Cav1.2 Ca^2+^ channels, a type of voltage-gated L-type Ca^2+^ channel, are ubiquitously expressed, and the predominant Ca^2+^ channel type, in working cardiac myocytes. Cav1.2 channels are regulated by the direct interactions with calmodulin (CaM), a Ca^2+^-binding protein that causes Ca^2+^-dependent facilitation (CDF) and inactivation (CDI). Ca^2+^-free CaM (apoCaM) also contributes to the regulation of Cav1.2 channels. Furthermore, CaM indirectly affects channel activity by activating CaM-dependent enzymes, such as CaM-dependent protein kinase II and calcineurin (a CaM-dependent protein phosphatase). In this article, we review the recent progress in identifying the role of apoCaM in the channel ‘rundown’ phenomena and related repriming of channels, and CDF, as well as the role of Ca^2+^/CaM in CDI. In addition, the role of CaM in channel clustering is reviewed.

## 1. Introduction

**Figure 1:**
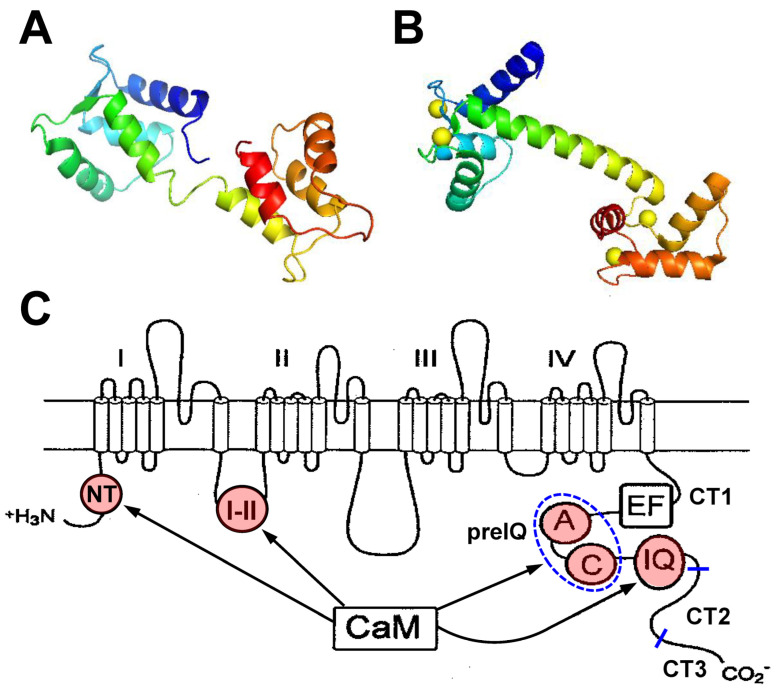
Structures of calmodulin (CaM) and the Cav1.2 Ca^2+^ channel. (**A**,**B**) apoCaM ((**A**), PDB:1CFD) and Ca^2+^/CaM ((**B**), PDB:1UP5) shown in rainbow color from the N-terminus (blue) to C-terminus (red) with Ca^2+^ indicated by the yellow spheres. (**C**) Schematic structure of pore-forming α1C subunit of Cav1.2 channel. CaM binding sites are indicated in red and labeled as follows: NT, NSCaTE domain; I–II, a site in I–II loop; (**A**,**C**) regions of preIQ domain; and IQ, IQ domain. The C-terminal tail is divided into CT1, CT2, and CT3 regions.

Calmodulin (CaM) was discovered in 1970 as a Ca^2+^ regulator, responsible for nucleotide phosphodiesterase in the brain [1]. Now, CaM is widely known as a ubiquitous eukaryotic Ca^2+^ sensor protein [2]. A small (16.7-kDa) dumbbell-shaped protein, CaM is ubiquitously distributed in tissues/cells and mediates various Ca^2+^-dependent signaling. CaM consists of an N-lobe, C-lobe, and linker connecting these two lobes (Figure 1). Each lobe has two EF-hand motifs that allow binding of two Ca^2+^ ions (four Ca^2+^ ions as a CaM molecule), thereby controlling various Ca^2+^-dependent pathways [3,4]. The two lobes have different affinities for Ca^2+^ and target binding. Affinity of the N-lobe for Ca^2+^ (Kd ~10 µM) is lower than that of the C-lobe (Kd ~1 µM), meaning that the dynamic range of CaM for Ca^2+^ spans a wide range, and allows it to transmit intracellular Ca^2+^ signals to more than 200 targets. CaM changes its conformation from a closed-in Ca^2+^-free state (apoCaM) to an open-in Ca^2+^-bound state (Ca^2+^/CaM) [5], forming the basis of target binding upon Ca^2+^ concentration ([Ca^2+^]) changes [6].

Voltage-gated calcium channels (VGCCs) are multimeric proteins (consisting of α1, β, and α2δ subunits; some with γ subunits) found in the membranes of nerve, muscle, and secretory cells [7]. VGCCs are divided into high-voltage-activated L-type (Cav1.1, Cav1.2, Cav1.3, and Cav1.4), P/Q-type (Cav2.1), N-type (Cav2.2), and R-type (Cav2.3) channels, because they are activated by relatively large depolarizations; as well as low-voltage-activated T-type (Cav3.1, Cav3.2, Cav3.3) channels, because they are activated by relatively small depolarizations. Among VGCCs, Cav1.2 is the dominant type in cardiac working muscle, while Cav1.3 and Cav3.2 are dominant in pacemaker (nodal) cells. Ca^2+^ influx through Cav1.2 channels is regulated by the negative feed-back mechanism, known as Ca^2+^-dependent inactivation (CDI), as well as the positive feed-back mechanism, known asCa^2+^-dependent facilitation (CDF) [8]. CaM is thought to play an important role in both CDI and CDF. Furthermore, CaM may have roles in channel trafficking and clustering/multimerization. Here, we review the regulation of cardiac Cav1.2 channels by CaM. Excellent reviews on similar subjects have appeared recently [8,9,10,11,12,13,14,15].

## 2. Functional Studies

### 2.1. Rundown of Channels

#### 2.1.1. History

‘Rundown’ of channels is a phenomenon in which the channel activity progressively declines under conditions where the intracellular environment is artificially changed. Rundown has been recognized since the 1970s, during the early period of intracellular recording of Ca^2+^ channels [16,17,18]. Rundown is thought to be caused by (1) channel dephosphorylation [17,19,20,21,22], (2) a switch-on of the inactivation process due to increased [Ca^2+^] [23], (3) a loss of activation factors [24,25,26,27,28], and/or (4) channel proteolysis [20,27,29] Thus, the rundown may be produced by multiple causes.

#### 2.1.2. How to Prevent the Rundown

To prevent the rundown of Ca^2+^ channels and achieve stable recording of channel activity, the ‘perforated’ patch-clamp method was developed [30]. This method does not break the patch membrane in the recording pipette, but instead applies ionophores, such as nystatin, to open small pores in the patch membrane. 

Another method to overcome the channel rundown is the replenishment of the factors required for the maintenance of Ca^2+^ channel activity. The rundown of Cav1.2 channels observed in inside-out patch-clamp mode is rapid and severe, with channel activity progressively decreasing until it disappears within several minutes [16,25,26]. Calpastatin, an inhibitor of Ca^2+^-activated protease calpain, was the first molecule reported to reverse the rundown [24,27,28]. Although this effect of calpastatin was first thought to be caused by the inhibition of calpain, its acute effect is localized to the non-inhibitory region of calpain [31,32]. Later, CaM (together with ATP, see below) was found to have the same effect [33]. This effect of CaM is independent of protein kinases, such as CaM-dependent protein kinase II (CaMKII), because it is not blocked by the non-specific protein kinase inhibitor K252a, or CaMKII inhibitors KN62, CaMKII peptide 281–309, and autocamtide-2-related inhibitory peptide [33]. Because calpastatin has a small effect compared to CaM and shows competition with CaM for its effect on Ca^2+^ channel activity, calpastatin is thought to act as a partial agonist for the CaM-binding site of the Cav1.2 channel [34,35]. 

### 2.2. Repriming of Channels

#### 2.2.1. CaM Is Crucial for Channel Activity

**Figure 2:**
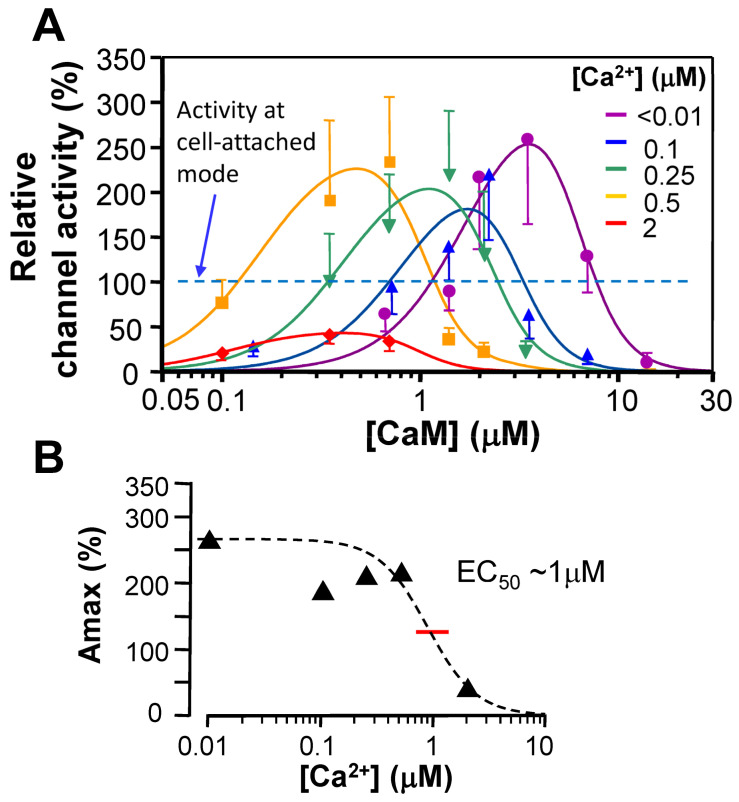
Effect of CaM in cell-free patches of cardiomyocytes. (**A**) Concentration-dependent effect of CaM on activity of Cav1.2 channels recorded in inside-out patches of guinea-pig cardiomyocytes at fixed [Ca^2+^], as indicated by colors on the right side of the graph. Channel activity was normalized to that recorded in the preceding cell-attached mode. Date are represented as the mean ± S.E.M. (n = 6–11) with regression curves with an equation as Amax · ([CaM]/Kd_f_)^nf^ / {1+([CaM]/Kd_f_)^nf^ } · 1/{1+([CaM]/Kd_i_)^ni^ }, where Amax is maximum channel activity, [CaM] is concentration of CaM, Kd_f_ and Kd_i_ are CaM’s dissociation constant for facilitation and inactivation, respectively, and n_f_ and n_i_ are the Hill’s coefficients for facilitation and inactivation, respectively. (**B**) Relationship between estimated maximum activity (Amax) and [Ca^2+^]. Data were fitted with Hill’s curve with EC_50_ ~1 μM (red bar). Reproduced from Han et al. [51] with permission.

CaM is thought to be crucial for Cav1.2 channel activity (see above). This view is in line with reports that indicate that CaM associates with both Cav1.2 and Cav2.1 channels to regulate their activity [36,37,38,39]. Mutations of the CaM-binding domain in the carboxy-terminal tail of the Cav1.2 channel α1C subunit disrupted binding of CaM to the channel and resulted in a loss of the regulatory effect of CaM [40,41]. The repriming effect of CaM (with 3 mM ATP at 80 nM Ca^2+^) on the Cav1.2 channels is concentration-dependent [half-maximal effective concentration (EC_50_) of ~1 µM] and had a maximum effect of 300–400% of the recordings in the preceding cell-attached patches. If we assume that the ascending phase of the dose-response relationship (Figure 2) simply reflects the binding of apoCaM to the channel, the Kd for CaM binding would be ~1 μM. This value is much higher than that reported for the binding of Ca^2+^/CaM to fragments of carboxy-terminal regions of the channel (10–100 nM) [38,42,43,44], but is lower than that reported for apoCaM binding to these fragments (~10 μM; [43,44]). Thus, these results suggest that the channel does not tightly interact with apoCaM at resting conditions ([Ca^2+^] ≈ 80 nM) but may be in dynamic equilibrium with free apoCaM in the cytoplasm near the internal side of the channel. CaM at ~0.3 μM + ATP (3 mM) produced channel activity that is comparable to that observed in the preceding cell-attached mode, suggesting that the concentration of free CaM near Ca^2+^ channels in myocytes was in this range. This concentration is comparable to that reported for cardiac myocytes, i.e., [CaM]_free_ value of 50–75 nM [45], considering an inhomogeneous distribution of CaM. We speculate that fewer than one-third of the channels are bound by apoCaM under resting conditions. Furthermore, CaM higher than 0.3 μM (+3 mM ATP) produced higher channel activity than that observed in the cell-attached mode. Collectively, these findings suggest a new mechanism of channel modulation, namely, changes in apoCaM concentrations following the release from or the absorption to CaM-binding proteins that modulate Ca^2+^ channel activity. Interestingly, both the individual N- and C-lobes of CaM can rescue channel activity from rundown, suggesting that CaM may reprime the Cav1.2 channel by binding to either lobe [46]. Notably, half-calcified CaM (Ca^2+^-bound only to C-lobe) bound to Cav1.2 channels with the C-lobe only (while the N-lobe is free) under resting conditions [see below (Section 3)] [47].

A similar effect of apoCaM was reported for Cav1.3 channels, which did not show a complete rundown in CaM-free conditions but had reduced activity, whereas, the application of apoCaM enhanced Cav1.3 channel activity [48,49]. However, the binding affinity of apoCaM for the Cav1.3 channel is reportedly only ~10 nM [50], meaning Cav1.3 channels are nearly saturated with apoCaM under physiological conditions near the channels (i.e., [CaM]_free_ = 50~500 nM) [45,51]. 

Numerous proteins interact with Cav1.2 channels [e.g., calpastatin, Ca^2+^-binding protein 1 (CaBP1), and α-actinin] [52], making them possible candidates for the ‘repriming’ proteins of these channels. In addition, Rad, an RGK protein that interacts with the β-subunit, suppresses Cav1.2 channel activity, e.g., [53]. Future studies are expected to identify the channel-repriming proteins other than CaM. 

#### 2.2.2. ATP Is Another Crucial Factor

The repriming effect of CaM requires the presence of ATP because neither CaM nor ATP alone can effectively restore channel activity in inside-out patches [25,26,33]. Because ATP can be partially substituted by AMP-PNP, a nonhydrolyzable ATP analog, a phosphorylation-independent mechanism is suggested (e.g., an ATP-binding site within or close to the channel) [54]. This view is further supported by the experiments that show that the effect of ATP can be partially replaced by other nucleotides with the following order of efficacy: ATP > GTP > UTP > ADP > CTP ≈ AMP [55]. Thus, the presumed nucleotide-binding site in the channel seems to favor a purine rather than pyrimidine base, and a triphosphate rather than a di- or monophosphate group. Notably, high concentrations (> 10 mM) of GTP, UTP, CTP, ADP, or AMP have inhibitory effects on channel activity [55]. Thus, protein phosphorylation is unlikely to be involved in the effect of ATP. A phosphorylation-independent action of ATP was previously reported [27,56]. Exploration for an ATP binding site in the Cav1.2 channel suggests that one occurs in the C-terminal tail of the α1C subunit [57]. Thus, the Cav1.2 channel is reprimed by CaM and ATP for activation upon depolarization. In fact, with CaM and ATP, activity of Cav1.2 channels can be stably recorded in inside-out patch-clamp mode for more than 1 h.

### 2.3. Slow Rundown of Channels

#### Contribution of CaMKII

CaM + ATP recovered Cav1.2 channel activity to 93% and 28% of that recorded in the cell-attached configuration, when applied 1 and 5 min after the excision of the patch from the cell, respectively, indicating time-dependent attenuation (called ‘slow rundown’). However, in the presence of 0.33 μM CaMKII, this attenuation was markedly retarded, showing 85% and 75% recovery when applied 1 and 5 min after the formation of the inside-out mode, respectively [58]. Although CaMKII T286D, a constitutively active mutant of CaMKII, effectively prevented a slow rundown similar to wild-type (WT), it failed to induce Ca^2+^-channel activity (only 2–10% of that recorded in cell-attached mode), thus allowing a ‘fast’ rundown to occur shortly after inside-out patch formation. These results suggest that CaMKII may not play a major role in reversing the fast rundown of channels, and that a large portion of CaMKII sites on the channels responsible for channel activity might already be phosphorylated in the basal condition. In pull-down assays, a GST-fusion protein containing amino acids 1509–1789 of the C-terminal region (CT1, Figure 1C) of guinea pig Cav1.2 treated with CaMKII, showed a higher affinity for CaM compared with CT1 treated with phosphatase. On the basis of this result, we proposed a model in which CaMKII-mediated phosphorylation of the Cav1.2 channel regulates its binding of CaM during the reversal of the rundown [59,60]. Furthermore, it is interesting to note that this effect might be related to the CaMKII-mediated facilitation (see below, Section 2.4.2).

Wang et al. found that CaMKII phosphorylates Thr1603 (guinea pig; Thr1604 in rabbit) within the preIQ domain of the C-terminal tail of the Cav1.2 channel [60]. A mutation of Thr1603 to Asp (T1603D) slowed the channel rundown in inside-out patch mode and abolished the time-dependent attenuation of CaM activity to reverse the rundown. These findings support the view that CaMKII-mediated phosphorylation of the proximal C-terminal (pCT) fragment (CT1) increases its binding with CaM, thereby maintaining the basal activity of the channel, while the dephosphorylation of CT1 showed an opposite effect. A similar effect of protein kinase A (PKA) phosphorylation of CT1 on the binding of CaM to CT1 has also been reported [61] (see also [22]).

### 2.4. Ca^2+^-Dependent Facilitation (CDF) 

#### 2.4.1. Overview of CDF

The Cav1.2 channel shows both voltage-dependent inactivation (VDI) and Ca^2+^-dependent inactivation (CDI). In addition, this channel shows both voltage-dependent facilitation (VDF) and Ca^2+^-dependent facilitation (CDF). CDI and CDF may involve CaM as a Ca^2+^ sensor [62]. Since the 1970s, the contractile force of the heart has been known to depend on its rate and pattern of stimulation [63]. An increase or decrease of contraction force in response to an increased pacing rate is called the positive or negative Bowditch effect or ‘staircase’ phenomenon, respectively, which are thought to primarily involve a change in Ca^2+^ release from the sarcoplasmic reticulum (e.g., [64]). 

Along with the positive staircase phenomenon of contraction force, a similar phenomenon has been observed in cardiac Ca^2+^ current induced by a train of repetitive depolarizations, or a strong depolarizing pre-pulse [65,66,67,68,69,70,71,72,73], a process termed Ca^2+^ current (I_Ca_) “staircase” or “facilitation”. Because the facilitation produced by a strong depolarizing pre-pulse is observed with charge carriers other than Ca^2+^ (e.g., Na^+^ or Ba^2+^), it is thought to be Ca^2+^-independent and thereby VDF. However, because the facilitation produced by repetitive depolarizations is not observed with Ba^2+^ as a charge carrier, it is called CDF. Accordingly, enhancement of I_Ca_ or I_Ba_ following a moderate increase of intracellular [Ca^2+^] in ventricular myocytes was reported [74,75,76,77,78]. Activity of L-type Ca^2+^ channels was potentiated by a moderate increase in intracellular [Ca^2+^] ([Ca^2+^]_i_, around 180–400 nM) and suppressed by a further increase in [Ca^2+^]_i_ (>600 nM) [77,78,79]. 

#### 2.4.2. CaMKII Mediated CDF

There are reportedly two types of CDF: CaMKII-mediated and CaMKII-independent. CaMKII-mediated CDF was independently reported by three groups [80,81,82]. All three studies reported that the pharmacological inhibition of CaMKII abolished CDF in mammalian cardiomyocytes.

Hudmon et al. reported that CaMKII is tethered to the C-terminus of α1C and mediates CaMKII-dependent CDF [83]. In contrast, Abiria et al. reported that CaMKII is anchored to the β2a subunit [84]. Multiple phosphorylation sites of CaMKII are reported in both α1C and β subunits. Specifically, CaMKII phosphorylates Thr498 (rat) of the β2a subunit, and then Ser439 (rabbit), Ser1517 (do.; corresponding to S1512 in a different isoform of rabbit), Ser1570 (do.), and Thr1603 (guinea pig) of the α1C subunit [60,85,86,87,88]. The cardiac myocytes of a knock-in mouse, in which S1512 (rabbit) and S1570 (do.) of Cav1.2 were mutated to alanine, showed no CDF, suggesting that these residues are phosphorylation sites responsible for CDF [88]. However, the physiological significance of the β2 subunit phosphorylation by CaMKII remains to be clarified [89]. It is of note that the CaMKII- mediated enhancement of I_Ca_ plays an important role in the development of isoproterenol-induced cardiac hypertrophy, and that the related gene expressions are significantly reduced by a peptide which inhibits binding of CaMKII to Cav1.2 channels [90,91].

Although CaMKII-mediated CDF of the native Cav1.2 channel is obvious in cardiac myocytes, CDF in in-vitro expression systems is observed only after CDI is masked by a mutation of Cav1.2 [41,42,92]. These findings suggest that unknown factors or additional mechanisms are involved in the manifestation of CDF. Yang et al. reported that cultured rat cardiac myocytes infected with adenoviral vectors harboring Ca^2+^-insensitive CaM mutant genes had increased I_Ca_ and were sensitive to CaMKII inhibitors, suggesting that the overexpression of Ca^2+^-insensitive CaM can activate CaMKII [93]. 

#### 2.4.3. CaMKII-Independent CDF

Other studies suggested that CDF is mediated by CaM, but independent of CaMKII [41,42,94]. Zühlke et al. showed that mutation of the IQ domain, a CaM-binding site in the C-terminus of Cav1.2, abolished CDI, and unmasked CDF [41,42] (see also [92]). Our investigation of the effects of CaM and CaMKII inhibitors on CDF and CDI with patch-clamp cell-attached recordings revealed that CDF and CDI were depressed and ultimately abolished by the CaM inhibitor chlorpromazine (1–100 μM) in a concentration-dependent manner. In addition, the CaM inhibitor calmidazolium (1 μM) produced a similar effect. In contrast, CaMKII inhibitors KN-62 (0.1–3 μM) and autocamtide 2-related inhibitory peptide (1 μM) significantly delayed the development of CDF and CDI without depressing either. We hypothesized that CaM is a key molecule for CDF and CDI, and that CaMKII plays a modulatory role in both [79].

#### 2.4.4. Biphasic Effects of CaM

Previous reports indicate the biphasic effects of [Ca^2+^]_i_ on I_Ca_, specifically increased I_Ca_ at low [Ca^2+^]_i_ and decreased I_Ca_ at high [Ca^2+^]_i_ [77,78]. These effects can be taken as a manifestation of CDF and CDI, suggesting that an interaction of apoCaM, or partially Ca^2+^-bound CaM, with the channel is necessary for its activation, and that the effect of Ca^2+^-saturated CaM may be Ca^2+^-dependent inactivation of the channel. Similar findings have been reported for the Cav1.3 channel [49] and ryanodine receptor (Ca^2+^-release channel in the sarcoplasmic reticulum) in skeletal muscle [95], whereby CaM is an activator at nanomolar [Ca^2+^] and an inhibitor at micromolar [Ca^2+^].

**Figure 3:**
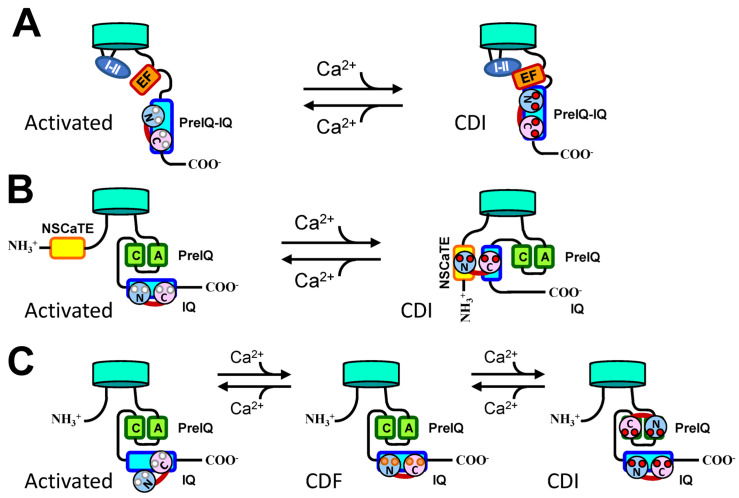
Models for CDI and CDF. (**A**) CDI model of Kim et al. [97]. Ca^2+^ binding to CaM, which is tethered to the preIQ-IQ region of the channel, induces a conformation change that initiates interaction with the EF-hand region, resulting in further interaction with the I–II loop, leading to CDI. (**B**) CDI model of Yue’s group [98]. Ca^2+^ binding to CaM induces bridging between the N- and C-termini, leading to CDI. (**C**) ‘Two CaM hypothesis’ [43,51,99]. At resting conditions, a fraction of channels are bound by CaM and the rest are CaM-free (low activity). (**A**) moderate increase in [Ca^2+^] increases the fraction of CaM-bound channels (high activity), leading to CDF. A further increase in [Ca^2+^] induces binding of the second Ca^2+^/CaM to the putative inactivation site, resulting in CDI. CaM’s N-lobe (light blue) and C-lobe (pink) with Ca^2+^-free (white circles) and partially Ca^2+^-saturated (pink circles) and Ca^2+^-saturated (red) EF-hand domains are illustrated. Other abbreviations are the same as those in Figure 1.

We examined the effects of CaM and Ca^2+^ on Cav1.2 channels in guinea pig ventricular myocytes in inside-out patch mode, whereby the channel rundown was controlled. At [Ca^2+^] of 0.1 μM, CaM (0.15–7.0 μM) + ATP (2.4 mM) induced channel activity with a bell-shaped relationship to [CaM], including a peak effect around 2 μM (Figure 2). Similar results were observed at a free [Ca^2+^] < 0.01 μM, or with the Ca^2+^-insensitive mutant CaM_1234_, suggesting that even apoCaM may be able to induce facilitation and inactivation of channel activity. The bell-shaped curve of CaM was shifted to a lower concentration (left) with increasing [Ca^2+^]. On the basis of these findings, a simple model for CaM- and Ca^2+^-dependent modulations of channel activity that assumes two CaM-binding sites was proposed (Figure 3). It suggests that both apoCaM and Ca^2+^/CaM can induce facilitation and inactivation of Cav1.2 channels, and that the basic role of Ca^2+^ is to accelerate CaM-dependent facilitation and inactivation [51]. We also investigated the concentration- and Ca^2+^-dependent effects of CaM mutants, CaM_12_ and CaM_34_, in which Ca^2+^ binding to N- and C-lobes is eliminated, respectively, on Cav1.2 channels by inside-out patch-clamping of guinea pig cardiomyocytes. Both CaM_12_ and CaM_34_ (0.7–10 µM) applied with 3 mM ATP produced channel activity after the rundown. Concentration–response curves were bell-shaped, similar to wild-type CaM, however, there was no obvious leftward shift of the curves with increasing [Ca^2+^], as observed for WT CaM, suggesting that both functional lobes of CaM were necessary for the Ca^2+^-dependent shift [96].

Reported [CaM]_free_ in resting cardiac myocytes (100 nM [Ca^2+^]) are 50–75 nM, representing only 1% of the total (free + bound to cellular constituents) [CaM] [45,100]. Therefore, CaM-bound Cav1.2 channels at the resting condition (i.e., [CaM]_free_ = 200 nM, considered a high [CaM] in Z-line area [45]) and [Ca^2+^] = 100 nM) represent approximately 10% of the total number of channels in the cell-free patches [51]. Thus, the potential dynamic range of the CaM-mediated CDF may be larger from a design point of view than considered thus far. Interestingly, the maximum effects of CaM_12_ and CaM_34_ were reduced as [Ca^2+^] increased [96]. A similar phenomenon was observed with WT CaM, suggesting that another Ca^2+^ sensor might exist in addition to CaM [51].

It is of note that the concentration of the intracellular Mg^2+^ shows biphasic effects on I_Ca_, facilitation and inactivation, similar to Ca^2+^ [101]. It may be interesting to explore if the Mg^2+^ effects are mediated by CaM.

#### 2.4.5. Mutations That Affect CDF

Numerous studies suggest that the IQ domain mutations in full-length channels can eliminate CDI and CDF [41,42,92,102,103]. Thus, it will be interesting to establish how CaM regulates VGCCs other than Cav1.2 through its interactions with their IQ motifs (reviewed elsewhere [11,15,104,105,106]. The Cav1.2 IQ domain residues F1618, Y1619, and F1622 (rabbit) are involved in the interaction with the N-lobe of Ca^2+^/CaM, and triple-alanine (TripleA) mutation of these sites eliminated CaM-mediated CDF [83,92], suggesting a hydrophobic interaction between the N-lobe of CaM and the IQ domain that is important for CDF. Kim et al. reported that the mutations in the preIQ-A (L1562A/F1563A, human α1C, 77) and the preIQ-C (W1593A and W1593E) together with I1624A (which unmasks CDF) reduced CDF, suggesting an important role for the Ca^2+^/C-lobe-preIQ interaction in CDF [107].

To date, there is no established CDF conformation of Cav1.2 and CaM. It is possible that multiple mechanisms and thus multiple conformations exist for CaM-dependent CDF of Cav1.2 channels. 

#### 2.4.6. Physiological Significance of CDF

As discussed above, CDF seems to involve at least two different mechanisms: one mediated by CaMKII phosphorylation, and one mediated directly by CaM. The latter CDF occurs rapidly in response to local [Ca^2+^]_i_ on a beat-to-beat basis, making Ca^2+^ influx “all-or-nothing” to ensure the reliability of excitation-contraction coupling. CDF mediated by CaMKII phosphorylation occurs more slowly (over several beats) and seems to have a different role. One study has proposed that the facilitatory mechanism may partly offset reduced Ca^2+^ channel availability at high heart rates (caused by increased CDI), contributing to the improvement of cardiac performance during exercise [108].

### 2.5. Ca^2+^-Dependent Inactivation (CDI)

#### 2.5.1. Overview of CDI

CDI was first demonstrated by Brehm and Eckert (1978) in voltage clamp recordings of the paramecium [109]. Eckert and collaborators also showed that the buffering of intracellular Ca^2+^ reduced the inactivation of I_Ca_, confirming the necessity for an increase of [Ca^2+^]_i_ for CDI [109,110,111]. CDI of Ca^2+^ channels was subsequently reported in various eukaryotic cell types across kingdoms, including placozoans, poriferans, ciliates, and vertebrates [112,113]. Among Cav channels in vertebrates, Cav1.2, Cav1.3, and Cav2.1 (P/Q type) channels show definitive CDI, while Cav1.1 shows modest CDI, Cav2.2 (N type) and Cav2.3 (R type) channels show tiny or undetectable CDI, and Cav1.4 and Cav3 (low-voltage activated type) channels show vestigial CDI [114,115]. 

The involvement of CaM in CDI is now widely accepted. Birnbaumer and colleagues found that the binding of CaM to the IQ domain of the C-terminus of the α1C subunit triggers CDI [116,117]. Reuter and colleagues [103,118] also reported that CDI of the Cav1.2 channel depends on the IQ motif of the C-terminus of the α1C subunit. Yue and collaborators also reported the involvement of CaM in CDI [102].

#### 2.5.2. Dephosphorylation vs. Ca^2+^/CaM

A mechanism other than the direct interaction of Ca^2+^/CaM has been suggested. Specifically, the Ca^2+^-dependent protein phosphatase calcineurin (CaN) can also accelerate the inactivation of the L-type Ca^2+^ currents during depolarization. Because CaN acts in a Ca^2+^/CaM-dependent manner, CaN-mediated dephosphorylation provides another molecular mechanism for CDI of Cav1.2 channels [119]. This hypothesis predicts attenuation or slowing of CDI by PKA phosphorylation, a phenomenon described for L-type currents in some neurons [119,120], but not Cav1.2-mediated currents in cardiomyocytes [12,121,122]. Because Cav1.2 channels are markedly upmodulated by PKA, the counteraction by responsible phosphatases should also be robust. If the time course of this action is slow, then the phosphatase effect would manifest as a down-modulation or slow CDI. 

#### 2.5.3. Mutations That Affect CDI

De Leon et al. reported that pCT regions containing EF-hand, preIQ, and IQ domains could transfer CDI from α1C to α1E, which had little CDI, suggesting that CDI was mediated by these domains [123]. Zühlke et al. explored important regions for CDI by investigating truncation mutants in the pCT of Cav1.2 channels [103]. They found three regions [i.e., the EF-hand domain, two hydrophilic residues (asparagine and glutamic acid) in the A region of preIQ, and IQ] that were important for CDI. Soldatov et al. revealed that IKTEG1572-76 in the preIQ-A region (human α1C,77) and LLDQV1600-04 in the preIQ-C region are important for CDI [124]. Qin et al. reported that the mutation of six residues in the IQ domain eliminated CDI [117], and Peterson found that a mutation replacing VVTL in the EF-hand domain (rabbit α1C) with the corresponding MYEM of α1E, which shows no CDI, eliminated CDI [125]. Furthermore, V1548Y (rabbit) mutation of the same region was enough to significantly reduce CDI. The authors suggested that this region might play a ‘transducer’ role rather than act as a sensor of CDI (see below 4.4. Downstream mechanism of CDI).

Since then, a number of mutation experiments around this area have been reported. Kim et al. reported that the mutation of TLF in the preIQ-A region abolished CDI [126]. It was confirmed that T1561A and L1565A in the preIQ-A (human α1C,77) reduced CDI [107], but not L1585A/L1588A in the dimer interface [127] nor W1593E in the preIQ-C affected CDI, thus confirming the importance of the preIQ-A region. In the same report, a W1593A mutation significantly reduced CDI [107], suggesting that the preIQ-C region was also important. Zühlke et al. reported that a I1624E (human α1C,77) mutation reduced the affinity of the IQ for CaM ~100-fold and eliminated CDI [42]. Erickson et al. reported that a IQ·FRK1654-60/5A (rabbit) mutation resulted in almost no CDI when WT CaM was co-expressed [128]. It has been reported that an I/E mutation in the IQ domain of Cav1.2 of transgenic mice abolished the regulation of CDI and CDF similar to findings of in vitro expression systems [129,130]. 

The importance of the pCT domain of the Cav1.2 channel for CDI is thus unequivocally documented. However, Dick et al. reported that the NT region also plays a role in CDI by bridging CaM between the NT and CT domains (NT-CT bridging model) [98]. The N-lobe of Ca^2+^/CaM interacts with a region named the N-terminal spatial Ca^2+^ transforming element (NSCaTE: ^81^SWQAAIDAARQAK^93^, rabbit), in which W82A, I86A, and R90A mutations significantly reduced CDI. Accordingly, whether the CT-restricted model or the NT-CT bridging model of CDI operates in intact myocytes remains an open question. It is possible that both mechanisms work simultaneously in myocytes. 

Mutations of CaM have been also reported to affect CDI (reviewed by Crotti et al. [131]). Among them, mutations of C-lobe are most frequently found, e.g., E141G [132].

#### 2.5.4. Interactions of Domains within C-Terminal Tail of Cav1.2

Hulme et al. reported that a domain immediately after IQ (termed the proximal C-terminal regulatory domain, PCRD) and a domain near the C-terminus (termed the distal C-terminal regulatory domain, DCRD) bind to each other, leading to a decrease in open probability [121]. Minobe et al. suggested a possible molecular mechanism by which the PCRD-DCRD interaction suppresses channel activity to disrupt the facilitatory effect of apoCaM and/or ATP on channel activity [133]. Crump et al. investigated the effects of the distal C-terminus of Cav1.2 (a.a. 1822–2171 of rabbit) on the activity of the channels and found that it inhibited I_Ba,L_ but not I_Ca,L_, and that its blockade was antagonized by Ca^2+^/CaM [134]. From these findings, they proposed a model in which the distal region functions as a reverse use-dependent inhibitor of the Cav1.2 channel. 

Lyu et al. reported a new interaction between the proximal (containing preIQ and IQ regions) and distal (distal of DCRD, a.a. 2116–2169 of guinea pig) regions of Cav1.2. Because binding is inhibited by Ca^2+^/CaM, the new distal interaction domain was named the “CaM-competitive domain” (CCD) [135]. Electrophysiological experiments showed that a CCD peptide inhibited, while a preIQ-IQ peptide facilitated, Cav1.2 channel activity in inside-out patches of guinea pig ventricular myocytes. These results suggest that the distal CCD inhibits the Cav1.2 channel by modulating its CaM-binding properties. The CCD overlaps with the distal ends of the C-terminal modulator (CTM) of Cav1.3 [136,137,138] and the inhibitor of CDI region of Cav1.4 [50,139,140], both of which are known to modulate CDI. The CTM of Cav1.3 has also been shown to compete with CaM for binding to the pCT region [50,138,141].

#### 2.5.5. C-Terminal Modulator of Cav1.3 and Cav1.4

Striessnig and colleagues found that an auto-modulatory domain within the CT of Cav1.3 and Cav1.4 precisely controls the gating properties of these channels [136,137,140,142,143]. This CTM, which consists of two α-helices in the PCRD and DCRD C-terminus, strongly reduces the CDI of channels and promotes their activation at more negative voltages [142]. Thus, the C-terminal splice variants of Cav1.3 show different CDI and activation properties [144]. Similar regulation mediated by the CTM has also been reported [139,145].

#### 2.5.6. Lobe Specific Effects of CaM

The N- and C-lobes of CaM appear to have distinct roles in CDF and CDI. For example, Ca^2+^-bound N-lobe produces CDI of Cav2.1 (P/Q type) channels, while Ca^2+^-bound C-lobe produces CDF [40]. For the Cav1.2 channel, CDI has only been attributed to the C-lobe of CaM [102,146,147]. However, Dick et al. observed CDI in HEK-293 cells expressing Cav1.2 and CaM_34_, which was abolished by the mutation of Trp82 to alanine in the N-terminal NSCaTE domain of the channel [98]. They proposed a model in which the C-lobe of CaM anchors to the C-terminus of Cav1.2, while the N-lobe of CaM binds to the N-terminus of the channel, bridging the two terminals to produce CDI. Ben-Johny and Yue summarized roles of the N- and C-lobes of CaM in sensing local or global [Ca^2+^] for Cav1 and Cav2 channels [11]. For the Cav1.2 channel, both lobes of CaM sense local Ca^2+^. However, it is difficult to believe that each lobe of CaM senses distinct local and global [Ca^2+^] because the distance between the two lobes is only ~1 nm. Thus, it may be better to consider these lobes in the temporal domain rather than in the spatial domain, with both decoding [Ca^2+^] in the same place with distinct temporal characteristics [148]; i.e., the C-lobe senses a rapid change of [Ca^2+^] with fast kinetics, while the N-lobe senses time-averaged [Ca^2+^] with slow kinetics [149].

Limpitikul et al. reported a mathematical model of CDI with decomposed effects of individual N- and C-lobes. This bi-lobal model of CDI indicates high cooperativity of both lobes in CDI [150]. Based on a study using nuclear magnetic resonance (NMR), it is proposed that the C-lobe of CaM selectively binds to its target (i.e., binds first to its target) [151], while the N-lobe binds afterwards, through an induced-fit or coupled conformational selection mechanism initiated by the C-lobe of CaM [152]. The idea that the C-lobe of CaM contributes to selectivity while the N-lobe contributes to flexibility is supported by recent molecular dynamics simulations [153].

#### 2.5.7. CaM-Dependent Inactivation

Han et al. found that even apoCaM and the Ca^2+^-insensitive mutant CaM_1234_ can induce inactivation of Cav1.2 channels at high concentrations, referred to as “CaM-dependent inhibition” [51]. These results imply that a change in [CaM] alone could affect Cav1.2 channel activity. In neurons, [CaM] is regulated by small neuronal IQ-motif proteins in response to cellular signals [154]. Recently, such proteins were also found in muscle cells [155]. Moreover, the modulatory effects of the growth-associated protein 43 (GAP43, neuromodulin) on ryanodine receptors and Cav1.1 channels have been reported [156]. Thus, it will be interesting to investigate whether such effects occur for Cav1.2 in cardiac myocytes. 

#### 2.5.8. Additional Ca^2+^-Sensing Site

At [Ca^2+^] higher than 0.5 μM, the repriming or facilitatory effect of CaM was attenuated with an apparent half-maximal inhibitory concentration of 0.9 μM, as if inactivation would have taken place. This result was not due to an inhibitory effect of Ca^2+^/CaM because this effect was also observed for the Ca^2+^-insensitive mutant CaM_1234_. Therefore, it is suggested that an ‘additional’ Ca^2+^ sensor for CDI exists other than CaM. Indeed, it is possible that Ca^2+^ interacts directly with the channels to promote a low-sensitivity CDI [51].

#### 2.5.9. CaBP1-Mediated Regulation

Numerous proteins interfere with CaM binding to pCT, including CaBPs, RGK proteins [157], α-actinin [44,158], and calpastatin [35]. Among these proteins, CaBP1 is most extensively studied. CaBP1 is a member of CaBP family, and highly expressed in the brain and retina [159]. CaBP1 suppresses CDI of Cav1.2 and brings about CDF [126,160]. CaBP1 binds to the IQ domain of Cav1.2 in a Ca^2+^-dependent manner (higher affinity with Ca^2+^ than without Ca^2+^) and competitively inhibits CaM binding to this domain [126,160,161,162,163,164]. However, CaBP1 also reportedly binds to the NT region, whose presence is required for a CaBP1-mediated inhibition of CDI [160].

CaBP1 produces a CaMKII-independent CDF by a different mechanism than a CaM-mediated CDF. Residues of F1618, Y1619, and F1622 in the IQ domain of Cav1.2 (human α1C,77) are involved in the interaction between the N-lobe of Ca^2+^/CaM and the IQ domain and play a role in CaM-mediated CDF of Cav1.2 [162,163]. A tripleA mutant of these residues eliminated CaM-mediated CDF [92] but not CaBP1-mediated CDF or the inhibition of CDI. Thus, Cav1.2 CDF mediated by CaM and CaBP1 have been suggested to use different molecular mechanisms.

CaM-mediated CDF of Cav1.2 was unmasked by a mutation of the IQ domain, I1624A [41,42,92]. However, co-expression of I1624A mutant channel with CaBP1 produced CDF with a magnitude indistinguishable from that of WT Cav1.2 with CaBP1, confirming that mechanisms of CDF mediated by CaM and CaBP1 are different. Findeisen et al. suggested that the N-lobe and interlobe linker of CaBP1 are important for inhibition of CDI and induction of CDF in Cav1.2 channels [161]. Findeisen et al. proposed a model in which the C-lobe of CaBP1 anchors to the IQ domain and the N-lobe of CaBP1 binds to the NT of Cav1.2 as a modulator [161,162], the latter being previously suggested [160]. Further details on regulation of Cav1.2 channels by CaBPs are described elsewhere [114].

The Cav1.3 channels in auditory inner hair cells show weak or no CDI, in contrast to the same channels in other tissues. Co-expression of CaBPs eliminates CDI of the Cav1.3 channel [165,166]. 

## 3. Molecular Biological Studies

### 3.1. Ca^2+^/CaM Binding Site

The binding of CaM to the IQ segment was first reported in the late 1990s [41,102,117]. This interaction is Ca^2+^-dependent with a 1:1 stoichiometry and occurs at Ca^2+^ concentrations above 100 nM. However, competition experiments with mutant CaMs defective in Ca^2+^ binding suggest that CaM is constitutively tethered to the channel [102]. The IQ motif and CaM are involved in both the facilitation and the inactivation of Cav1.2 [42]. Subsequently, several regions of the channel, including the NT, I-II loop, and preIQ (AC or CB domain) domains were identified [39,103,124,125,167,168,169].

Mouton et al. determined the Kd of the preIQ (CB domain) and IQ domains of Ca^2+^/CaM binding to be 12 and 40 nM, respectively [38]. They suggested that the ternary complex formed by preIQ, IQ, and CaM forms the switch leading to CDI. 

### 3.2. ApoCaM Pre-Association Site

Pre-association of apoCaM with Cav1.2 channels is proposed to function as a Ca^2+^ sensor for their Ca^2+^-dependent regulation [41,102,117]. Several regions of this channel, including the NT, I-II loop, preIQ (CB domain or AC region), and IQ domains, have been suggested as apoCaM-binding sites [39,103,124,125,167,168,169]. Among these, a region that includes both the preIQ and IQ domains in the C-terminal tail seem to be widely accepted as the pre-association site for CaM [41,102,117]. Furthermore, the IQ domain is required for both CDF and CDI [41,102]. Although the dual role of CaM in regulating CDF and CDI suggests multiple binding sites for CaM in the channel, a single CaM-binding site with multiple conformations depending on the Ca^2+^-binding state of CaM has also been suggested. Furthermore, phosphorylation mediated by Ca^2+^/CaM-dependent protein kinase II (CaMKII) may play a crucial role in CDF [170]. However, the precise molecular mechanisms for CDF and CDI remain to be clarified.

It is natural to postulate that Cav1.2 channels, the activity of which is modulated by CaM-sensed Ca^2+^, pre-associate apoCaM at low [Ca^2+^]_i_. This is the case for voltage-gated Nav1.2/1.5 channels, P/Q-type Ca^2+^ channels, the ryanodine receptor, Ca^2+^-activated K^+^ channels (SK and IK type), and the ether-a-go-go-type K^+^ channel (for review see [105,171,172,173,174]). Although the binding of Ca^2+^/CaM to Ca^2+^ channels, including the IQ domain, has been consistently demonstrated [41,102,117], the binding of apoCaM to channels was controversial in the early stage [39,102,117,167,168,175]. Later, Erickson et al. (2001; 2003) used fluorescence resonance energy transfer (FRET) experiments to provide clear evidence that apoCaM tethers to Cav1.2 channels near the IQ domain (< 100 Å) [128,176]. They raised two points for possible features of the pre-association: (1) pre-association of CaM could be coordinated by multiple segments in the proximal CT region, and (2) pre-association could require resting [Ca^2+^] for proper folding of a channel binding pocket for apoCaM [39,175]. Tang et al. reported a similar conclusion, namely that a peptide containing preIQ-C and IQ domains had the highest affinity to apoCaM [169]. 

Disruption of the binding site in the IQ domain for the N-lobe of Ca^2+^/CaM by simultaneous mutation of three N-lobe aromatic anchor residues (Phe1618, Tyr1619, and Phe1622 of human) to alanine eliminated CDF, suggesting that apoCaM tethers to the IQ domain [92]. This finding is supported by NMR showing that the C-lobe of apoCaM interacts with an IQ peptide [177]. Black et al. investigated the Ca^2+^ binding properties of CaM bound to IQ peptides of Cav1.2 and other types of Ca^2+^ channels [178]. They found that the binding of CaM to the IQ peptides increases the Ca^2+^ affinity of both CaM lobes. Evans et al. investigated the binding of apoCaM to the preIQ-A (a.a. 1588–1609 of rabbit), preIQ-C (a.a. 1614–1635), IQ (a.a. 1644–1670), and IQ’ (a.a. 1650–1675) regions [43]. Although Ca^2+^/CaM bound to all four peptides with low Kd values (≤2 nM), apoCaM bound to all four peptides with high Kd values (>10 μM). Among them, the IQ displayed a relatively high affinity (Kd ≈ 14 µM). These results suggest that apoCaM pre-associates with the IQ domain, whose N-terminal side (a.a. 1644–1649) is important for binding. 

Further evidence supporting the IQ region as an apoCaM-binding site is that the calpastatin L domain (CSL), which partially reprimes channel activity, and is thus thought to bind the apoCaM-binding site, competes with CaM for binding to the IQ domain [34]. Minobe et al. investigated the effect of competition between CSL and CaM on channel activity and found that CSL suppressed the channel-activating effect of CaM in a reversible and concentration-dependent manner [35]. Furthermore, CSL suppressed the binding of CaM to IQ in a competitive manner in a GST pull-down assay, however, it was later reported that preIQ also binds CSL [179]. Turner et al. reported the NMR structure of apoCaM bound to the IQ domain of Cav1.2. The C-lobe of CaM bound to Ile1654, Phe1658, and Lys1662 of the IQ domain (rat), while the N-lobe did not bind to IQ [44]. Thus, the possibility that the preIQ domain includes an apoCaM-binding site is not ruled out.

Black and Persechini investigated binding conformations between CaM and an IQ peptide with fluorescence experiments. When the C-lobe of CaM was Ca^2+^-free, it directed the N-lobe (probably Ca^2+^-free) to bind to a site within the IQ domain [180]. In contrast, when the C-lobe was Ca^2+^-bound, it directed the N-lobe (probably Ca^2+^-bound) to bind to a site upstream of the IQ domain, or a distant site. It will be interesting to determine how these conformations relate to those associated with CDF and CDI. 

Very recently, Bartels et al. reported that half-calcified CaM (Ca^2+^/C-lobe and apoN-lobe) promoted basal activity of the Cav1.2 channel [47] (see also [15]). This finding is based on their experimental observations that a CaM_34_ mutant, in which Ca^2+^-binding ability of the C-lobe is disabled, abolished the increasing effect on the open probability of Cav1.2 channels. They estimated that ~50% of Cav1.2 channels were bound to half-calcified CaM under basal conditions. In the future, it will be interesting to compare the effects of apoCaM and half-calcified CaM on Cav1.2 channel activity.

Notably, a mutation of the central linker of CaM, which connects the N- and C-lobes, markedly reduced apoCaM binding to preIQ and IQ domains, suggesting that the linker of CaM contributes to the binding of apoCaM to Cav1.2 [181].

### 3.3. Models of the Conformation of CDF and CDI

Zühlke et al. proposed a model for CDF and CDI in which apoCaM is bound to a site near the IQ domain at resting [Ca^2+^]_i_ and Ca^2+^ binds to the C-lobe of CaM at moderately increased [Ca^2+^]_i_, resulting in a conformational change that favors CDF [42]. At higher [Ca^2+^]_i_, fully-saturated Ca^2+^/CaM leads to further structural changes of the CaM-channel complex that favors CDI. Pitt et al. extended their hypothesis for Ca^2+^-dependent inactivation of L-type channels, stating that CaM is bound to preIQ-A and -C regions at resting Ca^2+^ levels, but upon the elevation of [Ca^2+^]_i_, the C-lobe of CaM shifts and binds to the IQ domain while the N-lobe remains anchored on the preIQ-A domain [39]. This hypothesis suggests that the binding of a single CaM to sites in the CT domain of Cav1.2 causes conformational changes of the channel depending on the Ca^2+^-bound condition of CaM. Similar models with different details were proposed by Tang et al. (2003) [169] and Erickson et al. (2003) [128].

Kim et al. proposed a model in which three regions of Cav1.2 (i.e., CaM-binding IQ domain, EF-hand motif, and I-II intracellular loop) are involved [126]. Specifically, the CaM-binding IQ domain of α1C transmits the CDI signal via the α1C EF hand motif to the putative pore-occluder, the α1C I-II intracellular linker [62,126]. Ile1654 is important for this transmission of the CDI signal from IQ to EF domains because mutation of I1654A abolished CDI [97]. CDF might be produced by burying the Ile of the IQ motif and preventing its interaction with the EF hand [62]. Ames et al. [15] and Yadav et al. [99] proposed a similar model of CDI in which the C-lobe of apoCaM, or half-calcified CaM (Ca^2+^/C-lobe and apoN-lobe), interacts with the IQ domain while the N-lobe is free, allowing IQ to also interact with the EF-hand domain in the pCT. This conformation prevents interactions between the EF-hand domain and the inactivation gate in the III-IV linker of Cav1.2 (instead of I-II linker, see above), maintaining the channel in an activatable state. When [Ca^2+^]_i_ is elevated, the N-lobe of fully calcified Ca^2+^/CaM interacts with the IQ domain, making the EF-hand domain release IQ and instead engage with the III-IV linker, leading to inactivation of the channel. Thus, this model suggests that Ca^2+^ binding to the N-lobe of CaM to IQ is critical to trigger CDI (IQ switch model). 

We proposed the “Two CaM” hypothesis in which one CaM binds to the first high-affinity (facilitation) site of the channel to promote repriming and CDF, while the other CaM binds to the second low-affinity (inactivation) site of the channel to promote CDI [51]. This model explains why even apoCaM can produce channel inactivation when a high enough concentration is applied. Asmara et al. [182] and Minobe et al. [35] extended this hypothesis by suggesting that apoCaM dynamically binds to the IQ or preIQ-IQ regions to reprime the channel for opening upon depolarization. When [Ca^2+^]_i_ is elevated, the first CaM binds with Ca^2+^ and transforms the channel to a state that favors CDF, whereas the second Ca^2+^/CaM binds to the preIQ domain to complete CDI. 

For Cav2.1 channels, Catterall and Few proposed a CDF and CDI model in which local rises in [Ca^2+^]_i_ lead to Ca^2+^ binding to the C-lobe of CaM in a pre-associated site, causing translocation to the IQ domain and CDF [183]. Further rises in [Ca^2+^]_i_ causes CaM to be fully liganded and interact with both IQ and CBD (=preIQ) domains to produce CDI. 

A quite different model involving the N-termini of Cav1 channels has also been proposed [98]. In this model, the N-lobe of Ca^2+^/CaM binds to a N-terminal region (NSCaTE) in a Ca^2+^-dependent manner to bridge the N-terminus (N-lobe of Ca^2+^/CaM bound) and C-terminus (C-lobe of Ca^2+^/CaM bound) of Cav1.2 and Cav1.3 channels, thereby accelerating their closure [98,148]. This model is supported by other groups, e.g., Liu & Vogel reported the NMR solution structure of a Ca^2+^/CaM-N lobe-NSCaTE peptide [184]. The authors pointed out that Ca^2+^/CaM acts almost like an “adaptor” protein to bridge the N- and C-terminal domains of the channel. Simms and Zamponi proposed a similar model in which apoCaM tethers to the preIQ/C (PCI-2)-IQ region and, upon [Ca^2+^] elevation, the C-lobe of Ca^2+^/CaM moves to a more proximal C-terminal region (PCI-1), and the N-lobe binds to the N-terminus of the Cav1.2 channel, thereby producing CDI [106]. 

Although CaM binds to the N-terminus of Cav1.2, a mutation in NSCaTE of Cav1.2 (Trp82, Ile86, and Rrg90 to TripleA) abolishing the binding of CaM only slightly weakened the CDI [185]. The authors subsequently reported that the NT and pCT domains directly interact, regardless of whether pCT binds with apoCaM, and Ca^2+^/CaM reduces this interaction. On the basis of these findings, they claim that dissociation of the NT-CT interaction leads to CDI, proposing a model in which the closed state of the channel configures the NT-CT interaction with apoCaM bound to CT; this interaction is dissociated in the open state until a second Ca^2+^/CaM binds to NT during CDI [186]. Furthermore, although preIQ and IQ motifs are reportedly conserved in all L-type channels of metazoans, the NSCaTE motif is missing in some arthropod species and lower animals, such as platyhelminths and cnidarians. Regardless, these species show clear CDI, suggesting that CDI mediated by NSCaTE is an optional mechanism developed later in the evolutionary process [113,187]. 

Kawaji et al. [188] and Minobe et al. [189] carried out similar experiments and found that even with CaM_1234_-linked Cav1.2 channels, externally applied Ca^2+^/CaM could induce CDI and that NT-deleted channels failed to support CDI. From these results, they suggest that two types of CDI might coexist, one produced by two CaMs at CT, and the other produced by one CaM with NT-CT bridging. 

For Cav1.3 channels, Ben-Johny et al. proposed a model of CDI based on systematic Ala mutagenesis of the entire C-terminal tail of the channel [190]. In this model, apoCaM pre-associates with the IQ domain through the C-lobe, and a hot-spot between the EF-hand and preIQ domain by the N-lobe. Upon [Ca^2+^] elevation, the N-lobe of Ca^2+^/CaM departs to the N-terminal NSCaTE, while the C-lobe of Ca^2+^/CaM moves to the EF-preIQ region. This model is similar to the one that was proposed by Simms and Zamponi [106], however, small differences exist between these two models, including the N-lobe of apoCaM binding to a region upstream of the preIQ domain in Cav1.3, but to preIQ in Cav1.2. Although the sequence between the EF-hand domain to preIQ (^1555^QPPLG-RTALR^1601^, rabbit Cav1.2) shows high homology between Cav1.2 and Cav1.3, CaM binding to this region of Cav1.2 has yet to be verified. 

Overall, models for CDF and CDI remain controversial. Specifically, the contributions of one or two CaMs, and whether regulation of CDI is limited to CT or involves both NT and CT remain to be elucidated. Further studies are needed to reach unequivocal conclusions. 

### 3.4. Downstream Mechanism of CDI

The molecular basis of mechanisms downstream of CDI has also been investigated. There are three hypotheses for the “endpoint” mechanism: (1) the hinged-lid theory states that a cytoplasmic inactivation particle plugs the pore, analogous to Nav channels and Shaker type Kv channels [191]; (2) collapse of the selectivity filter (SF) occurs, similar to C-type (or P-type) inactivation of K channels; and (3) allosteric inhibition of an intracellular activation gate occurs by a mechanism distinct from that of VDI [75,97,161,192,193,194,195,196]. Tadross et al. investigated the molecular endpoints of CDI by systematically mutating the intracellular S6 segments of all repeats (I to IV) of the Cav1.3 channel [197,198]. They found that allosteric inhibition of the intracellular activation gate is most likely involved in CDI, while a hinged-lid with a shield (that repels lid closure) mechanism is implicated in VDI; this idea is supported by others in Cav1.2 as well as Cav1.3 [199,200,201,202]. Although Abderemane-Ali et al. [203] suggest that the N-terminal cytoplasmic region [98,148,167,190] and Cavβ/I-II loop complex [97,161,200] affect CDI, how conformational changes in these cytoplasmic regions lead to CDI remains unsolved [97,161,196,197,204,205]. Interestingly, Guggenheimer et al. (2016) have proposed a hypothesis that Ca^2+^/CaM-induced changes in the NT-CT interaction may, in part, accelerate CDI of CaV1.2 [186]. 

Other studies reported a close link between ion selectivity and CDI, based on the properties of CDI and SF being simultaneously affected by the mutations of SF or changes in extracellular [Ca^2+^] [193,206], and the observation that Gd^3+^ blockade of SF appears mutually exclusive with inactivation [204]. The existence of an SF gate and inner gate formed by the S6 helices [186,197,198,199,207,208] suggests that VGCCs have two gates to regulate their activity. Thus, the molecular endpoint of CDI remains an open question. In addition, whether CDI and VDI share common pathways [97,161,195], or act by independent mechanisms [196,198], needs to be addressed. 

### 3.5. CaMKII Phosphorylation Sites

Phosphorylation of the β2a subunit responsible for CaMKII-mediated facilitation of Cav1.2 channels has been observed [87], suggesting that this phosphorylation modulates the interaction between the β subunit and the C-terminal tail of the α1 subunit, thereby manifesting CDF [87,209,210]. However, a site in the I-II loop (rabbit Ser439) and two sites in the CT1 domain (rabbit Ser1517 and 1575) are functionally phosphorylated by CaMKII [85,86,88], where the roles of these sites in Ca^2+^-dependent regulation are currently unclear. 

We investigated a hypothetical CaMKII phosphorylation site on Cav1.2 that contributes to channel regulation and found that CaMKII phosphorylates Thr1603 (guinea pig, Thr1604 in rabbit) within the preIQ domain of the C-terminal tail of the guinea pig Cav1.2 channel. Mutation of T1603D slowed channel rundown in inside-out patch mode and abolished the time-dependency of CaM effects to reverse rundown. We also found that CaMKII-mediated phosphorylation of the pCT fragment (CT1) increased, while dephosphorylation of CT1 decreased its binding with CaM. These findings suggest that CaMKII regulates the binding of CaM to the Cav1.2 channel, thereby maintaining its basal activity [60].

### 3.6. Two CaM Hypothesis

Ca^2+^/CaM binds to multiple peptides derived from α1C of the Cav1.2 channel [98,160,167,169,190], in order of decreasing affinity, IQ > preIQ > I-II loop > N-terminal peptide [35,160,182]. Early studies reported that the binding of CaM to IQ and CB (preIQ) domains is competitive, suggesting only one CaM can bind to the C-terminus [168,169,211,212]. Mori et al. reported that one CaM per channel was sufficient to produce CDI for Cav1.2 channels covalently fused with a single CaM molecule [211]. Furthermore, live-cell FRET analysis of interactions between CaM and Cav1.2 channels suggest a 1:1 CaM/channel ratio [213].

A more recent study showed that a peptide containing both preIQ and IQ domains (Leu1599–Leu1668) bound with approximately 2 molecules of CaM per peptide, supporting the hypothesis that two CaM molecules can simultaneously bind to the C-terminus of the Cav1.2 channel, and modulate its facilitatory and inhibitory activities [182]. Two CaM molecules were simultaneously bound to the C-terminus of the Cav1.2 channel in crystallographic studies [107,127,214], and the Two CaM model is supported by other studies [35,43,51,186,215,216]. Evans et al. investigated the binding of CaM to preIQ and IQ domains by monitoring the increases in the fluorescence anisotropy of fluorescein-conjugated C-terminal peptides of Cav1.2 [43]. They found that Ca^2+^/CaM bound to both preIQ and IQ domains favorably (Kd ≤ 2 nM), proposing a CDI model in which the first CaM binds to IQ and the second CaM binds to preIQ by making an A-C hairpin formation that bridges the A and C regions. Notably, both preIQ-A and C regions are reportedly important for CDI (see ‘Mutations that affect CDI’ above) [107]. 

At high [Ca^2+^], 2 molecules of Ca^2+^/CaM bind to Cav1.2 channels [217]. Chakouri examined whether single or multiple CaMs are involved in Ca^2+^-dependent regulation of VGCCs using two CaM or Ca^2+^-insensitive CaM mutants (CaM_1234_) tethered to the α and/or β subunit [218]. They found that VGCCs respond to signaling from the α-subunit-tethered CaM and concluded that Ca^2+^ regulation depends on the pre-association of a single CaM with the α-subunit. However, Minobe et al. found that when the Gly linker connecting CaM with the Cav1.2 channel was short (<12 a.a.), the binding of CaM was so strong that externally applied secondary CaM could not produce its effect on Cav1.2 [189]. Thus, it is possible that the use of a longer Gly linker could produce a different conclusion.

### 3.7. ATP-Binding Site (s)

Feng et al. examined ATP binding to the Cav1.2 channel using a photoaffinity method [57]. A photoactive biotin-conjugated ATP [8-azido-2′/3′-O-(2-aminoethyl-carmamoyl)-ATP-biotin] labeled both the proximal C-terminal region (a.a. 1509–1789 of guinea pig) and N-terminal region (a.a. 6–140) of the Cav1.2 channel. Interestingly, ATP labeling of the Cav1.2 channel was enhanced by the presence of CaM (~4 fold with CaM: Cav1.2 = 1:1 compared with that in the absence of CaM). Thus, it is possible that CaM and ATP synergistically affect Cav1.2 channel activity. 

## 4. Structural Studies

Van Petegem et al. reported the crystallographic structure of a Ca^2+^/CaM-bound IQ domain (a.a. 1611–1644 of human α1C,77) showing the complex with a parallel orientation and binding governed mainly by hydrophobic interactions. Phe1618, Tyr1619, and Phe1622 interact with the Ca^2+^/CaM N-lobe, whereas Tyr1627, Phe1628, and Phe 1631 interact with the Ca^2+^/CaM C-lobe [92]. Isothermal titration calorimetry analysis revealed two Ca^2+^/N-lobe binding sites (Kd ≈ 50 nM and Kd ≈ 20 μM) and a single high-affinity Ca^2+^/C-lobe site (Kd ≈ 2 nM) in the IQ domain. The high-affinity site of the N-lobe overlaps with that of the C-lobe. Fallon et al. reported a similar crystal structure analysis for a complex of Ca^2+^/CaM and IQ peptide (a.a. 1665–1685 of human Cav1.2, Q13936) [219]. Because the Ile1672 residue (thought to form an intramolecular interaction that induces CDI) was buried, they suggested that this form might be associated with CDF. 

**Figure 4:**
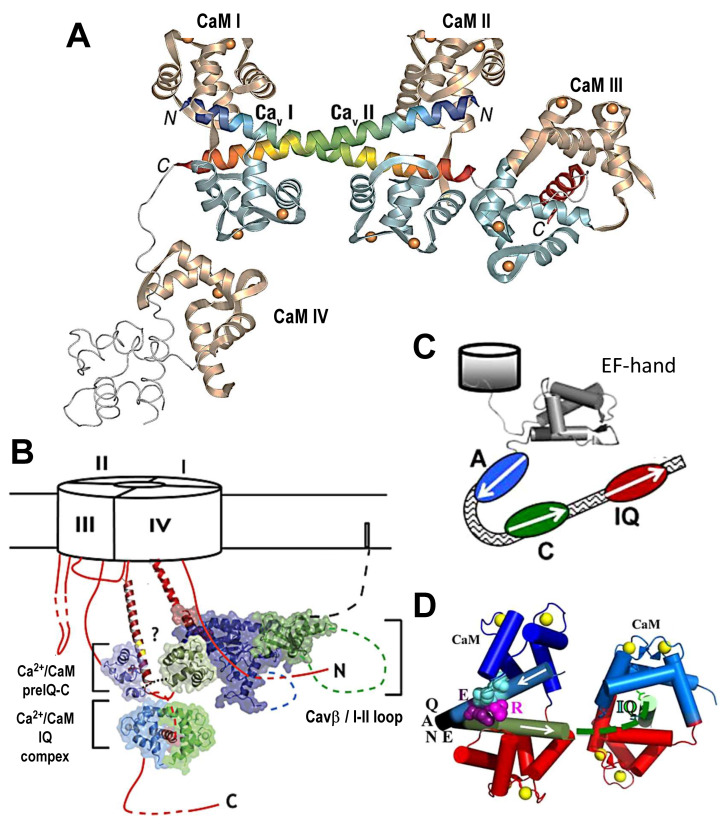
Structure models of preIQ-IQ/Cav1.2 bound with CaM. (**A**) Crystallographic structure of two preIQ-IQ bound with four CaM. CaM N-lobes, tan; C-lobes, light blue; Ca^2+^, gold spheres. The preIQ domain (I and II) are blue to orange rainbow starting from the N termini (N); IQ domain of Ca_V_ I is red. The preIQ-IQ loop regions, IQ domain of Ca_V_ II and C-lobe of CaM IV are computer modeled (thin light gray lines). From [127] with permission. (**B**) Structure model of preIQ-IQ of Cav1.2 bound with two CaM. The ‘?’ signifies the potential that the second Ca^2+^/CaM anchors at the C region of preIQ to interact with other components of Cav1.2. Cavβ (green and blue) bound to I–II loop (red) are shown as a candidate of such components. Orientation of the C-terminal tail relative to the other intracellular components of Cav1.2 is not identified. From [107] with permission. For the second CaM, the C-lobe is bound to preIQ, and the N-lobe binds another region, possibly the Cavβ/I–II loop. (**C**,**D**). Structure model of preIQ-IQ of Cav1.2 bound with two CaM. The preIQ structure is assumed to bend or turn at ^1610^QANE^1613^ region (rabbit) between the A and C regions (**C**) to allow the second CaM to bridge these regions to form a tertiary complex (**D**). The R^1601^ and E^1605^, which interact with both the N− and C-lobes of CaM are also shown. The model is based on PDF:3G43. From [43] with permission.

Fallon et al. extended the CaM-binding peptide to a preIQ-IQ peptide and explored its CaM-binding activity [127]. They found that four CaM molecules bound to two preIQ-IQ peptides, raising the possibility of Cav1.2 channel dimerization (Figure 4A). Kim et al., however, reported that the channel formed monomers but not dimers in Xenopus oocytes transfected with Cav1.2 and Cavβ_2a_, and the stoichiometry of Ca^2+^/CaM: CT was 2:1. Furthermore, they suggested that an interaction between the Ca^2+^/C-lobe and preIQ C-region is involved in CDF [107] (see also [104]).

## 5. Channel Clustering/Multimerization

Synchronized openings of multiple Cav1.2 channels have been reported [220]. These findings indicate that CaM antagonists (e.g., W7 and CaM inhibitory peptide), activators of protein kinase Cα, or a specific Cav1.2 channel mutation (associated with Timothy syndrome) that moves CaM away from the IQ domain in the C-terminal tail of the channel promoted coupled gating between Cav1.2 channels [220].

Fallon et al. reported the crystal structure of Cav1.2, which contains an anti-parallel dimer of preIQ helices bridged by two Ca^2+^/CaMs (see above) [127]. However, sedimentation equilibrium experiments using purified Ca^2+^/CaM:Cav1.2 preIQ–IQ complexes suggested a model of 2:1 stoichiometry [107]. Furthermore, the measurement of the discrete photobleaching steps of a Cav1.2 a1 subunit bearing a green fluorescent protein tag in its C-terminal revealed that most channels (>95%) bleached in a single step, suggesting that most channels exist in monomeric form [107,221].

In contrast, Dixon et al. demonstrated that Cav1.2 channels form functional couplings through C-terminal-to-C-terminal interactions, mediated by Ca^2+^/CaM binding to the preIQ domain in native ventricular myocytes and an in vitro expression system [222]. The same group reported that β-adrenergic stimulation promotes surface expression of a sub-sarcolemmal pool of Cav1.2 channels and formation of channel “superclusters”, thereby enhancing channel co-operability [223]. 

## 6. Trafficking of Cav1.2 Channels

Trafficking of membrane proteins, such as Cav channels, includes several processes: (1) exportation from the endoplasmic reticulum; (2) transportation to the cell surface; (3) stabilization in the plasma membrane; and (4) internalization, degradation, and recycling to the plasma membrane [224,225,226]. For Cav1.2 channels, these processes are regulated by the intrinsic properties of isoforms of the α1 subunit of the channel, the associated auxiliary subunits, and the regulatory proteins such as CaM.

Ca^2+^/CaM reportedly binds to GDP-bound inactive RGK proteins and subsequently promotes transformation of RGK to the GTP-bound, Ca^2+^/CaM-released activated form, which antagonizes binding of Cavβ to the Cavα1 subunit by competitive binding of Cavβ to RGK [227,228,229,230,231]. Thus, the overall effect of Ca^2+^/CaM is the inhibition of Ca^2+^ channel cell-surface expression. However, activity induced Ca^2+^/CaM (but not apoCaM) reportedly accelerated the trafficking of channels to the neuronal cell surface [232]. In addition, overexpression of Ca^2+^-insensitive CaM_1234_ accelerated the trafficking of channels to the surface of cultured cells, similar to native CaM [233]. Gao et al. reported that the surface expression of mutant Cav1.2 channels lacking the preIQ3 region (i.e., preIQ-C region) was completely abolished, despite the presence of the β2a subunit, suggesting that the binding of CaM to the channel is required for its surface expression [234], see also [232,235]. Furthermore, reports suggest that α-actinin binds to the IQ domain and stabilizes Cav1.2 at the surface membrane, and its displacement by Ca^2+^/CaM induces Ca^2+^-dependent endocytosis of these channels [158,236]. Thus, the role of CaM in trafficking of Cav1.2 channels remains controversial and further studies are required.

## 7. Conclusions and Perspectives

Many questions about the regulation of Cav1.2 channels by CaM remain to be answered. For example, CaM and other intracellular proteins that interact with channels, such as CaBP1 and α-actinin, may contribute to channel repriming [163]. In the future, it will be interesting to determine whether these proteins can prevent rundown of Cav1.2 channels in inside-out patches. In addition, CDF seems to be mediated by various mechanisms, including one mediated by the direct CaM interaction with channels and other mechanisms not related to the direct interaction of CaM and the channel. Reportedly, CaM can indirectly influence channel activity through activation of CaMKII, phosphodiesterase type 1, or calcineurin, for reviews see [237]. In particular, the CaMKII-mediated phosphorylation of channels enhances their activity, contributing to CDF, therefore, each mechanism should be analyzed independently. For CDI, multiple conformations of the channel-CaM complex in its inactivated state can exist, i.e., bridging of the N- and C-terminals by CaM and a conformation formed by two CaMs at the preIQ-IQ region of the C-terminus. From this viewpoint, solving the structure of the intracellular regions of the Cav1.2 channel would be an important achievement. Finally, although a role for CaM seems plausible for the clustering and trafficking of Cav1.2 channels, more studies are required to reach a clear conclusion.
